# Traditional Chinese medicine herbal mixture LQ arrests FUCCI-expressing HeLa cells in G_0_/G_1_ phase in 2D plastic, 2.5D Matrigel^®^, and 3D Gelfoam^®^ culture visualized with FUCCI imaging

**DOI:** 10.18632/oncotarget.2983

**Published:** 2015-03-12

**Authors:** Lei Zhang, Chengyu Wu, Michael Bouvet, Shuya Yano, Robert M. Hoffman

**Affiliations:** ^1^ AntiCancer, Inc., San Diego, CA 92111, USA; ^2^ Department of Traditional Chinese Medicine Diagnostics, Nanjing University of Traditional Chinese Medicine, Nanjing 210029, China; ^3^ Department of Surgery, University of California at San Diego, San Diego, CA, USA; ^4^ Department of Gastroenterological Surgery, Okayama University Graduate School of Medicine, Dentistry and Pharmaceutical Sciences, Okayama, Japan

**Keywords:** TCM, herbal mixture, LQ, paclitaxel, FUCCI

## Abstract

We used the fluorescence ubiquitination-based cell cycle indicator (FUCCI) to monitor cell cycle arrest after treatment of FUCCI-expressing HeLa cells (FUCCI-HeLa) with a traditional Chinese medicine (TCM) herbal mixture LQ, previously shown to have anti-tumor and anti-metastatic activity in mouse models. Paclitaxel was used as the positive control. In 2D monolayer culture, the untreated control had approximately 45% of the cells in S/G_2_/M phase. In contrast, the LQ-treated cells (9 mg/ml) were mostly in the G_0_/G_1_ (>90%) after 72 hours. After treatment with paclitaxel (0.01 μm), for 72 hours, 95% of the cells were in S/G_2_/M. In 2.5D Matrigel^®^ culture, the colonies in the untreated control group had 40% of the cells in S/G_2_/M. LQ arrested the cells in G_0_/G_1_ after 72 hours. Paclitaxel arrested almost all the cells in S/G_2_/M after 72 hours. In 3D Gelfoam^®^ culture, the untreated control culture had approximately 45% of cells in G_2_/M. In contrast, the LQ-treated cells were mostly in G_0_/G_1_ phase (>80%) after 72 hours treatment. Paclitaxel resulted in 90% of the cells arrested in S/G_2_/M after 72 hours. The present report suggests the non-toxic LQ has potential to maintain cancers in a quiescent state for long periods of time.

## INTRODUCTION

Traditional Chinese medicine (TCM) compositions usually comprise multiple herbs and many components may be are necessary for efficacy. We previously compared the efficacy of the TCM herbal mixture LQ against lung cancer in mouse models with doxorubicin (DOX) and cyclophosphamide (CTX). LQ was effective against primary and metastatic lung cancer without weight loss and organ toxicity. In contrast, although CTX and DOX had efficacy in the lung cancer models, they caused significant weight loss and organ toxicity. LQ also had anti-angiogenic activity as observed in lung tumors growing in nestin-driven green fluorescent protein (ND-GFP) transgenic nude mice, which selectively express GFP in nascent blood vessels. LQ also increased survival of tumor-bearing mice, comparable to DOX. *In vitro*, lung cancer cells were killed by LQ, comparable to cisplatinum. LQ selectively killed cancer cell lines compared to normal cell strains unlike cytotoxic drugs which killed both similarly [1].

In another previous study, LQ significantly inhibited pancreatic cancer tumor growth and metastasis in orthotopic models with no overt toxicity. LQ also increased survival of tumor‐bearing mice. The antitumor efficacy of LQ was comparable with gemcitabine (GEM), but with less toxicity than GEM [2].

Sakaue-Sawano et al. [3] developed a fluorescence ubiquitination-based cell-cycle indicator (FUCCI). With FUCCI, G_0_/G_1_ cells express an orange-red fluorescent protein and S/G_2_/M cells express a yellow-green fluorescent protein. We previously determined with FUCCI imaging the cell-cycle phase of invading cancer cells [4] and the spatial-temporal cell-cycle phase distribution of cancer cells within tumors before and after chemotherapy [5].

The present report uses FUCCI imaging to demonstrate the effect of LQ on the cell cycle arrest of HeLa cells.

## RESULTS AND DISCUSSION

### Effect of LQ or paclitaxel on the cell-cycle phase distribution of FUCCI-HeLa cells growing in 2D culture

**Figure 1:**
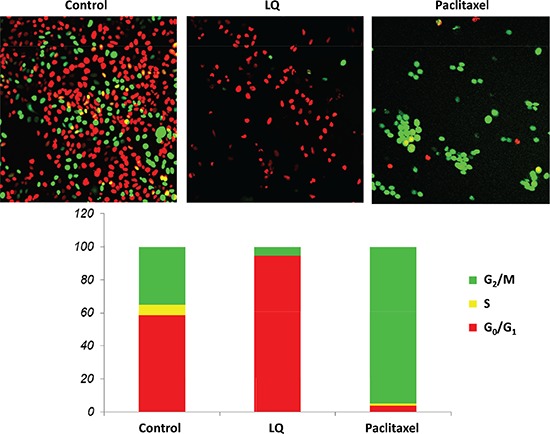
LQ or paclitaxel treatment of FUCCI-HeLa cells growing in 2D culture G_0_/G_1_ arrest of HeLa FUCCI cells was observed with LQ (9 mg/ml) treatment. Untreated control cells were distributed throughout the cell cycle. Paclitaxel was used as the positive control and resulted in an S/G_2_/M block. Bar graphs show the distribution of the cell-cycle phases with each treatment or control. See Materials and Methods for details.

Untreated control HeLa-FUCCI cells exhibited a combination of orange-red and yellow-green nuclei. Approximately 60% of the cells were in S/G_2_/M and 40% in G_0_/G_1_. The cultures were approaching confluence. After treatment with LQ (9 mg/ml) for 72 hours, the HeLa-FUCCI cells were almost all arrested in G_0_/G_1_. Only approximately 5% were in S/G_2_/M phases and the remainder in G_0_/G_1_. After treatment with paclitaxel (0.01 μM), for 72 hours, approximately 5% of the cells were in G_0_/G_1_ with the rest of the cells arrested in S/G_2_/M. Almost all the paclitaxel-treated cells fluoresced yellow-green, in contrast to LQ-treated cells, which almost all fluoresced orange-red (Figure 1).

### Effect of LQ or paclitaxel on the cell-cycle phase distribution of FUCCI-HeLa cells growing in 2.5D Matrigel culture

**Figure 2:**
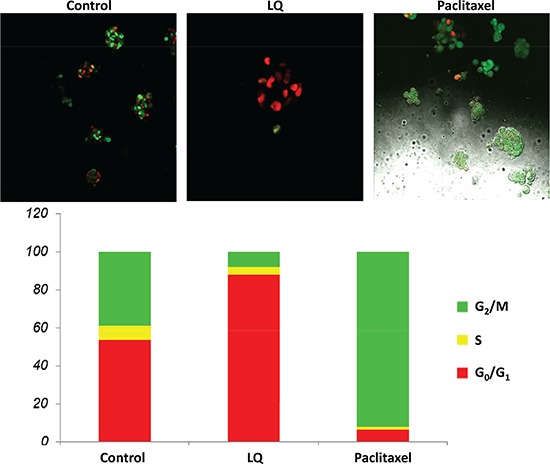
LQ or paclitaxel treatment of FUCCI-HeLa cells growing in 2.5D Matrigel culture G_0_/G_1_ arrest of HeLa-FUCCI cells was observed with LQ (9 mg/ml) treatment. Untreated control cells were distributed throughout the cell cycle. Paclitaxel was used as the positive control and resulted in an S/G_2_/M block. Bar graphs show the distribution of the cell-cycle phases. See Materials and Methods for details.

The untreated control cells exhibited a combination of orange-red and yellow-green nuclei with the cells growing in colonies. Approximately 50% of the cells were in S/G_2_/M and 50% in G_0_/G_1_. LQ (9 mg/ml) had a similar effect in Matrigel as on plastic, with most of the cells arresting in G_0_/G_1_ and only 10% in S/G_2_/M after 72 hours of treatment. Paclitaxel (0.01 μM) in 2.5 D Matrigel culture, as in 2D culture, arrested the cells almost all in S/G_2_/M after 72 hours treatment (Figure 2).

### Effect of LQ or paclitaxel on the cell-cycle phase distribution of FUCCI-HeLa cells growing in Gelfoam^®^ histoculture

**Figure 3:**
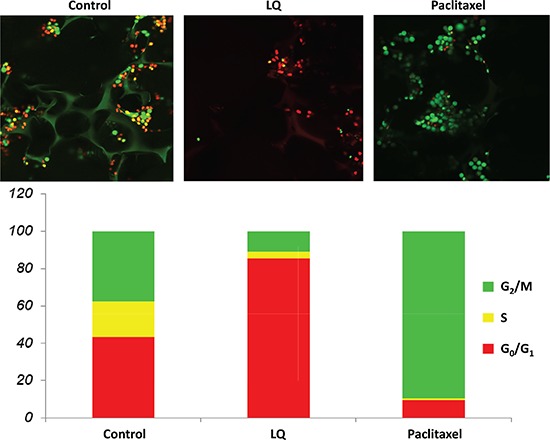
LQ or paclitaxel treatment of FUCCI-HeLa cells growing in Gelfoam® histoculture G_0_/G_1_ arrest of HeLa-FUCCI cells was observed with LQ (9 mg/ml) treatment. Untreated control cells were distributed throughout the cell cycle. Paclitaxel was used as the positive control and resulted in an S/G_2_/M block. Bar graphs show the distribution of the cell-cycle phases. See Materials and Methods for details.

Control cells in Gelfoam^®^ histoculture exhibited a combination of orange-red and yellow-green nuclei. Approximately 60% of the cells were in S/G_2_/M with almost 20% in M, a surprisingly large percentage. Treatment with LQ (9 mg/M) for 72 hours resulted in over 80% of the cells being arrested in G_0_/G_1_, similar to 2 D plastic culture and 2.5 D Matrigel culture. Paclitaxel (0.01 μM) resulted in 90% of the cells arrested in S/G_2_/M, as in 2D and 2.5D culture, after 72 hours treatment (Figure 3).

Methionine deprivation, either by nutritional means or recombinant methioninase (rMETase) treatment, selectively trapped cancer cells in S/G_2_ [6, 7]. Excess thymidine, or its analogs, arrested cancer cells in S-phase [8–10]. The calcium-channel blocker mibefradil arrested glioblastoma cells at the G_1_/S checkpoint [11]. Statins, such as Lovastatin, can arrest cancer cells in G_0_/G_1_ [12, 13]. PDO332991, a pyridopyrimidine-selective inhibitor of cyclin-dependent kinases 4 and 6, induced early-G_0_/G_1_ arrest in various cancer cell types, including melanoma and breast cancer *in vitro*, and in cancer xenograft models. RO-3306, also a cyclin-dependent kinase inhibitor, reversibly arrested 95% of treated cells in G_2_ phase [14–17]. Since LQ has been shown to be non-toxic, it has potential to maintain cancers in a quiescent state for long periods of time.

Previously-developed concepts and strategies of highly-selective tumor targeting [18–29] can take advantage of cell-cycle imaging of cancer cells and G_0_/G_1_ blockage by LQ, described in the present report.

## MATERIALS AND METHODS

### Cell line

FUCCI-expressing HeLa cells were grown in DMEM supplemented with 10% fetal bovine serum.

### Establishment of HeLa cells stably transfected with FUCCI plasmids

Plasmids expressing mKO2-hCdt1 (yellow-green fluorescent protein) and mAG-hGem (orange-red fluorescent protein) (Medical and Biological Laboratory, Nagoya, Japan) [3], were transfected into HeLa cells with Lipofectamine™ LTX (Invitrogen).

### Two-dimensional monolayer culture

FUCCI-expressing HeLa cells were grown as monolayer in 35 mm dishes in DMEM supplemented with 10% fetal bovine serum.

### Matrigel (2.5 D culture)

Matrigel (100 μl/chamber well) was pipetted into the center of the well and then allowed to spread evenly for 15–30 minutes at 37°C in order for the Matrigel to solidify. FUCCI-expressing HeLa cells (4 × 10^4^) were grown for 4 days in Matrigel and DMEM supplemented with 10% fetal bovine serum.

### Gelfoam^®^ (3D culture)

Sterilized Gelfoam^®^ was cut and placed in wells. The Gelfoam^®^ was hydrated in DMEM medium for 24 hours. A pellet of HeLa-FUCCI cells (10^6^) in 20~30 μl DMEM supplemented with 10% fetal bovine serum, was gently placed on top of the Gelfoam^®^. Medium was added to the same height as the Gelfoam^®^.

### Preparation of crude extracts of chinese herbs

LQ consists of a mixture of the following Chinese medicinal herbs: *Sinapis alba, Atractylodes macrocephala, Coix lacryma-jobi*, and *Polyporus adusta* (School of Pharmacy, Nanjing University of Traditional Chinese Medicine, Nanjing, China) as previously reported [30]. The ratio of the herbs used was (2:3:4:3). Dried plant parts used were: *Sinapis albe*, seed; *Atractylodes macrocephala*, root; *Coix lacryma-jobi*, kernel; *Polyporus adusta*, whole mushroom. Each herb was extracted in boiling water for 20 mins, the solution was filtered and the residue extracted with 75% ethanol when filtered. The water extract and ethanolic extract were combined and concentrated by lyophilization. The lyophilized powder was suspended in PBS and the mixture was vortexed for 1 min and incubated at 80°c for 30 min., cooled to room temperature, and was then centrifuged at 2000 rpm for 10 min. The supernatant was collected at a final concentration to 90 mg/ml and diluted and filtered through a 0.2 mm membrane for *in vitro* use [31].

### Chemotherapy drugs

Paclitaxel (Taxol) (Bedford Laboratories, Bedford, OH) was prepared by diluting to 0.2 μM as a stock solution.

### Imaging of FUCCI-expressing HeLa cells

Confocal laser scanning microscopy was performed with the FV1000 confocal laser scanning microscope (Olympus Corp., Tokyo, Japan) with 2 laser diodes (473 nm and 559 nm). A 4× (0.20 numerical aperture immersion) objective lens and a 20× (0.95 numerical aperture immersion) objective lens (Olympus) were used. Scanning and image acquisition were controlled by Fluoview software (Olympus). The tracing data were imported to Velocity 6.0 version (Perkin Elmer), where all 3D analysis was performed [32].
